# Vacuum/Compression Valving (VCV) Using Parrafin-Wax on a Centrifugal Microfluidic CD Platform

**DOI:** 10.1371/journal.pone.0058523

**Published:** 2013-03-11

**Authors:** Wisam Al-Faqheri, Fatimah Ibrahim, Tzer Hwai Gilbert Thio, Jacob Moebius, Karunan Joseph, Hamzah Arof, Marc Madou

**Affiliations:** 1 Medical Informatics and Biological Micro-electro-mechanical Systems Specialized Laboratory, Department of Biomedical Engineering, Faculty of Engineering, University of Malaya, Kuala Lumpur, Malaysia; 2 Department of Electrical Engineering, Faculty of Engineering, University of Malaya, Kuala Lumpur, Malaysia; 3 Department of Biomedical Engineering, University of California Irvine, Irvine, United States of America; 4 Department of Mechanical and Aerospace Engineering, University of California Irvine, Irvine, United States of America; 5 Ulsan National Institute of Science and Technology, World Class University, Ulsan, South Korea; Texas A&M University, United States of America

## Abstract

This paper introduces novel vacuum/compression valves (VCVs) utilizing paraffin wax. A VCV is implemented by sealing the venting channel/hole with wax plugs (for normally-closed valve), or to be sealed by wax (for normally-open valve), and is activated by localized heating on the CD surface. We demonstrate that the VCV provides the advantages of avoiding unnecessary heating of the sample/reagents in the diagnostic process, allowing for vacuum sealing of the CD, and clear separation of the paraffin wax from the sample/reagents in the microfluidic process. As a proof of concept, the microfluidic processes of liquid flow switching and liquid metering is demonstrated with the VCV. Results show that the VCV lowers the required spinning frequency to perform the microfluidic processes with high accuracy and ease of control.

## Introduction

Centrifugal microfluidic CD platforms offer many advantages over larger traditional fluidic platforms such as a reduction of the required sample/reagent volumes, portability, low fabrication cost, and full automation. In one of its simplest embodiments, a microfluidic CD platform controls fluid sequencing based on the balancing of the centrifugal force and the capillary force [1]. Examples of applications developed on the centrifugal microfluidic CD platform include enzyme linked immunosorbent assays (ELISA) [2], [3], real time polymerase chain reaction (PCR) [4], [5], and particle separation [6]–[8].

The most essential of mechanisms on a microfluidic CD is a valve that allows for fluid flow sequencing. A valve is a component that stops (normally-open valve), starts (normally-closed valve) or controls (proportional valve) fluid flow through a specialized passage or channel [1], [9], [10]. According to Oh et al [9], microfluidic valves fall under two main categories: passive (dependent on centrifugal forces) and active (independent of centrifugal forces) valves. Many kinds of valves fall under these two categories such as mechanical, non-mechanical, and externally actuated valves. These valves were categorized according to the mechanism of operation and/or actuation methods. For a valve to function effectively in a diagnostic process, several requirements must be met. First, it must be able to operate with relevant clinical samples and reagents of widely varying physicochemical properties typically used in diagnostic processes [1], [10], [11]. The valves must be unaffected by these substances to prevent the degradation of the valve before its actuation. Second, for all the steps of a traditional clinical diagnostic process to be replicated identically on a microfluidic CD platform [12] may require a multitude of valves including passive and active valves, proportional valve, normally-closed valves, and normally-open valves. Third, fluid manipulation for any diagnostic process must be tightly controlled. Failure to adhere to any of these requirements will result in rejection by the FDA and misdiagnosis of a disease or error in clinical test results, especially when biomarkers are present in very low concentrations. In general, the main criteria for a successful microvalve includes the prevention of evaporation or leakage sample, reduction of the dead volume, short time to actuation, and reduced power consumption [9].

While there are a wide variety of passive valves available, such as hydrophobic, hydrophilic, siphon, Coriolis, flap valves [1], [10], [12]–[16] etc, in most cases, these valving techniques lack a physical barrier to prevent evaporation of liquids during test storage and operation [16]. Furthermore, there are serious challenges involved in making passive valves repeatable and manufacturable. To meet the requirements for a diagnosis process, active valves are often required alone or in addition to passive valves.

Active valves on the CD platform are components that operate independently from the centrifugal force. These valves require external forces to actuate a physical blocking mechanism for liquids and vapors, examples include; pneumatic valve, ice valves, wax valves, hydrogel valves, and mechanical base valves [4], [17]–[24]. However, these valves are either too complicated to fabricate, too expensive, limited to individual actuation, or may involve the mixing of valve material with the analyte. Among active valves, wax valves provide the benefit of addressing the issue of liquid evaporation, are relatively simple to fabricate and actuate and represent the least expensive option. Moreover, by implementing wax material with different melting temperature, different valves can be actuated at different instances in the same process [19]. Wax valves implemented today however, come with two obvious disadvantages, i.e., the unnecessary heating of sample and reagents that are in close proximity to the valve [19], and the possible contamination of the sample/reagent as the wax is mixed with the liquid after actuation [18], [19].

To solve the issues of unnecessary heating and possible contamination, we have introduced and implemented a new Vacuum/Compression Valve (VCV) by using paraffin wax to seal chamber venting channels/holes. The new method has two main advantages: relocating the point of heating away from the diagnostic process, and preventing the direct contact between the samples and the wax material.

To implement VCVs, paraffin wax is installed on the venting channels/holes of the source and destination chambers. The VCV causes a vacuum state in source chambers, and air-compression in destination chambers. The vacuum/air-compression state prevents the fluid from flowing out of the chamber. In this work, we show how a VCV is implemented, and demonstrate that a VCV provides proper valving and sealing of a sample/reagent containing chamber.

## Centrifugal Force

In a microfluidic CD, the centrifugal pressure (*P_centrif_*) is the main force that propels the liquid from the center toward the rim of the microfluidic CD during spinning. This force can be calculated by the following equation [25]:
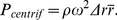
(1)


At a certain rotational speed in *rpm*, fluid starts flowing from the source chamber to the destination chamber. This speed is referred to as the “burst frequency” and it can be calculated using the following equation [25]:
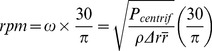
(2)


In this study, Equations 1 and 2 are utilized to calculate the theoretical burst frequency to be compared to the experimental results.

## Methodology

### 1. Experimental Setup and Materials

**Figure 1:**
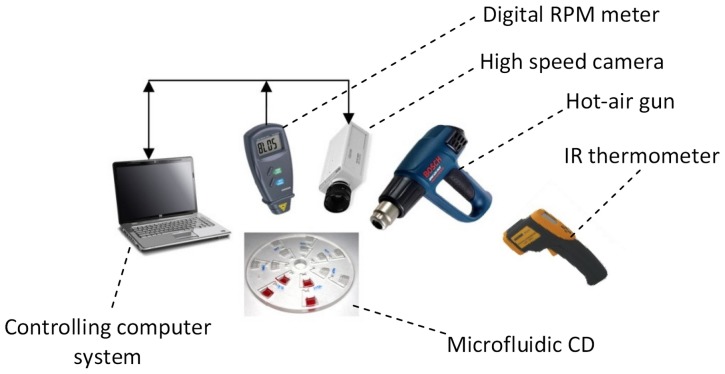
Experimental Setup. Experimental setup: controlling computer system connected to high speed camera, digital rpm meter, manually controlled forced convection heatingand IR thermometer.

A custom made computerized CD Spin Test System equipped with a high speed camera and an rpm measuring laser is used to run the tests (see figure 1). For melting the wax during actuation of the valve, an industrial hot-air gun is positioned 1 cm above the top surface of the CD to provide forced convection heat transfer. The hot-air gun is equipped with nozzle of 1 cm diameter to focus the forced convection heat only on the required area. This nozzle provides a heating zone of 1 cm^2^ on the CD surface which is enough to cover only the wax-plug area. The CD surface temperature is measured with a digital infrared (IR) thermometer every 90 seconds.

**Figure 2:**
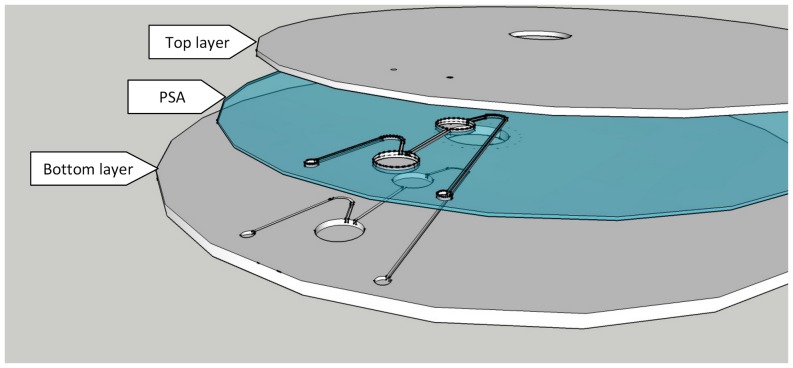
Microfluidic CD layers. Three layers microfluidic CD: 2 mm thick PMMA as the top layer, and 4 mm thick PMMA for bottom layer, PSA layer in the middle.

The micro-scale features (channels and chambers) of the CD fluidic platform are engraved in a 4 mm thick Polymethyl methacrylate (PMMA) plastic layer (bottom layer) using a Computer Numerical Control (CNC) machine (model VISION 2525, by Vision Engraving and Routing Systems, USA). A 2 mm PMMA layer with venting holes cut through is fabricated as a cover layer (top layer) for the microfluidic CD. The PMMA layers are then bonded together using a Pressure Sensitive Adhesive (PSA) material (by FLEXcon, USA) (see figure 2). A cutter plotter (model *PUMA II*, by GCC, Taiwan) is utilized to cut the microfluidic CD design in the PSA layers. Channels and chambers, corresponding to the design of the bottom PMMA layer, are cut out from the PSA layer to avoid having the liquid come in contact with the adhesive material of the PSA layer. This ensures a more consistent solid-liquid interface between the liquid and the channel/chamber walls.

Paraffin wax with a melting temperature of 57.2°C (135°F) is applied for the normally-closed and normally-open VCV valves. A custom made press-roller machine is used for bonding the different CD layers together. De-ionized (DI) water with red food dye (at a ratio of 1 part dye to 10 part water) is used as the test liquid.

### 2. Expermental Method and CD Design

In this study, the effect of vacuum/compression valving on the burst frequency is investigated. Paraffin wax is applied to the venting holes of source/destination chambers to create vacuum/compression valving respectively. The following experiments were conducted for this study: microfluidic CD heat profiles are registered, vacuum/compression valve effectiveness is measured, and two possible applications for the VCV valving method are demonstrated.

#### 2 (a) Microfluidic CD heat profile

First, a study was conducted to investigate the heating profile for the microfluidic CD to better understand the thermal behavior. As shown in figure 2, three-layer microfluidic CDs (2 PMMA plastic layers and one PSA layer) were fabricated to perform this study. Forced convection heating at 130°C is applied to the top surface of the CDs. The CD was spun at different speeds of 0 to 350 rpm, and the CD surface temperature was measured using a digital IR thermometer. This experiment allows for the determination of the surface temperature required to melt the wax valves at different rotation speeds.

#### 2 (b) Vacuum/Compression valve (VCV) effectiveness

**Figure 3:**
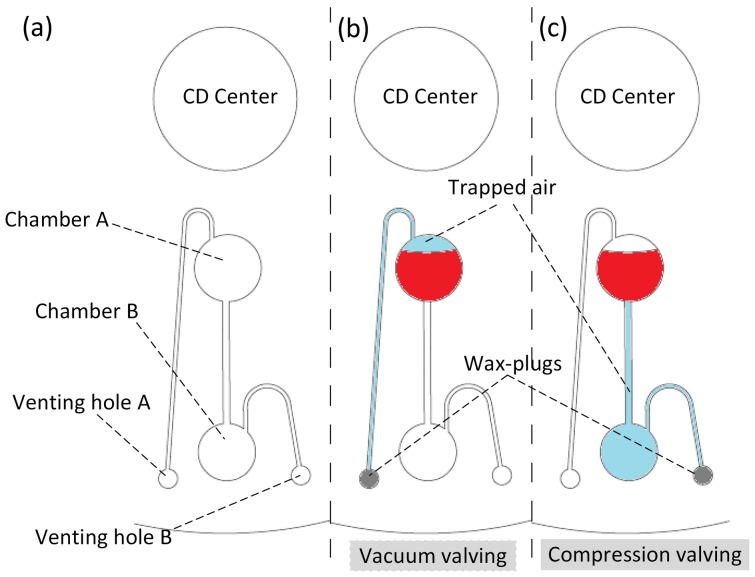
VCV microfluidic CD Design. CD Design for test of vacuum/compression valve effectiveness. (a) general design (b) vcuum valving experimental setup, (c) compression valving experimental setup.

In this study, the same three-layer microfluidic CD design was used to fabricate and test the proposed VCV valving method. Figure 3(a) shows the design of a VCV valve fabricated with a set of source and destination chambers. As illustrated, the design consists of a source Chamber A, destination Chamber B, and the corresponding Venting hole A and B. Figure 3(b) & (c) illustrates the vacuum and compression setup using the proposed basic design. To actuate vacuum valving, venting hole A is sealed with a wax plug to create air-trapping on top of the liquid in source chamber A (Figure 3(b)). This trapped air prevents liquid in source chamber A to move toward destination chamber B until the venting hole A is released (by melting the wax plug). On the other hand, Figure 3(c) shows the setup for compression valving. Venting hole B is sealed by a wax plug to create air-trapping in the channel below the liquid in source chamber A and also in chamber B. This trapped air stops liquid from chamber A to flow to chamber B until venting hole B is released. In both vacuum and compression cases, increase the CD spin speed increases the vacuum pressure on top of the liquid (Figure 3(b)), and increase the air compression pressure in destination chamber B (Figure 3(c)). This experiment tests the effectiveness of the vacuum and compression states by respectively sealing venting hole A & B with wax plugs. The CD is spun and the wax is melted with forced convection heating. The results of the two experiments are compared with a control set, and theoretical calculation obtained by using Equations (1) and (2).

#### 2 (c) Applications of VCV

Many potential microfluidic processes can be controlled using the proposed VCV.

Vacuum valving can be implemented to replace the traditionally used passive valves in a multi-step microfluidic process. The bursting of each chamber can be redefined by selectively melting the various wax plugs and releasing the venting holes of the respective source chambers. Compression valving can be implemented in liquid flow switching and liquid metering applications.

**Figure 4:**
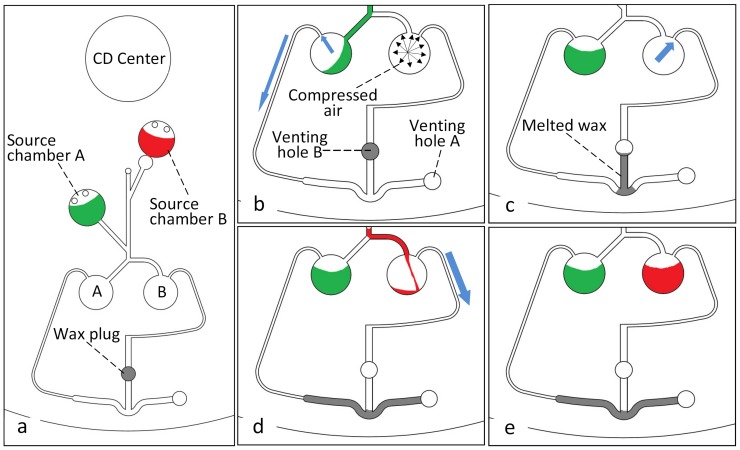
CD design for Switching process. Microfluidic CD design for liquid switching, (a) position of chambers and wax plug, (b) sealed venting B forces liquid to go to chamber A, (c) melting of wax-plug releases venting hole B and blocks venting hole A, (d) liquid goes to chamber B, (e) final liquid status.

Figure 4(a) shows the design of the microfluidic CD fabricated to perform a liquid flow switching process. The design consists of two source chambers (A & B), two destination chambers (A & B), and the corresponding venting holes with compression wax plugs (venting hole A & B, see figure 4(a)). Two different colored DI water aliquots (red and green) are used to allow for a clearer observation of the switching process. A 40 µl volume of the two colored DI water is injected in each one of the source chambers which are designed to have different burst frequencies. In this process, a VCV incorporating both a normally-closed and a normally-open compression valve (see figure 4(b)) is used to switch the liquid flow direction to the intended destination chamber. Figure 4 (b, c, d, and e) illustrates how the switching design is expected to work. It can be observed from figure 4(b), that air compression in chamber B (created by the sealing of venting hole B) prevents liquid from flowing into destination chamber B, and forces the liquid to burst into destination chamber A. (Note the air in chamber B is only compressed when liquid from chamber A attempts to flow into destination chamber B. The principle behind this occurrence is discussed in detail in the “Results and Discussion” section). Afterward, the wax-plug is melted and the centrifugal force pushes it towards the U bent junction, effectively blocking the venting channel leading to venting hole A. Air compression now occurs in destination chamber A, and the next bursting of liquid will be forced into destination chamber B. The main advantage of this design is that a single wax plug is used to block the two venting holes in two different steps.

**Figure 5:**
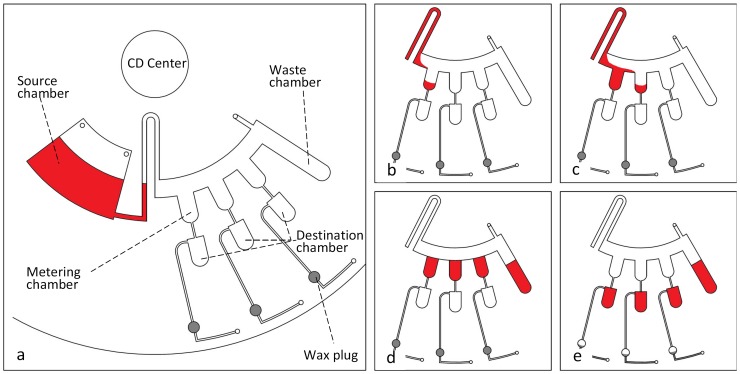
CD design for liquid metering process. Microfluidic CD design for liquid metering, (a) position of chambers and wax plugs, (b) liquid fills the first metering cahmber, (c) liquid fills the second metering chamber, (d) liquid filled all metering chambers and extra liquid flows to the waste chamber, (e) melting of wax-plug allows liquid to move to the destination chambers.

Another microfluidic process implemented in this study using the proposed VCV is liquid metering. The microfluidic CD designed to perform this process is presented in figure 5(a). The design consists of three metering chambers that are respectively connected to three destination chambers via 0.4 µm-width channels. The venting holes of the three destination chambers are controlled with the proposed VCV (air compression valve). Figure 5(b, c, d, and e) presents the expected sequence of liquid metering process which starts with the pumping of the colored DI water to fill the metering chambers during the spinning process. The liquid fills the metering chambers, but does not enter the destination chambers because of the air-compression created by the VCV. After the liquid settles and levels in the metering chambers, the venting holes are released by melting the wax plugs and the liquid then flows into the destination chambers.

## Results and Discussion

This work is divided into three parts: microfluidic CD heat profile, VCV effectiveness test, and the two possible microfluidic processes using the proposed VCV. The following subsections present and discuss the results of each part separately.

### 1. Microfluidic CD Heat Profile

**Figure 6:**
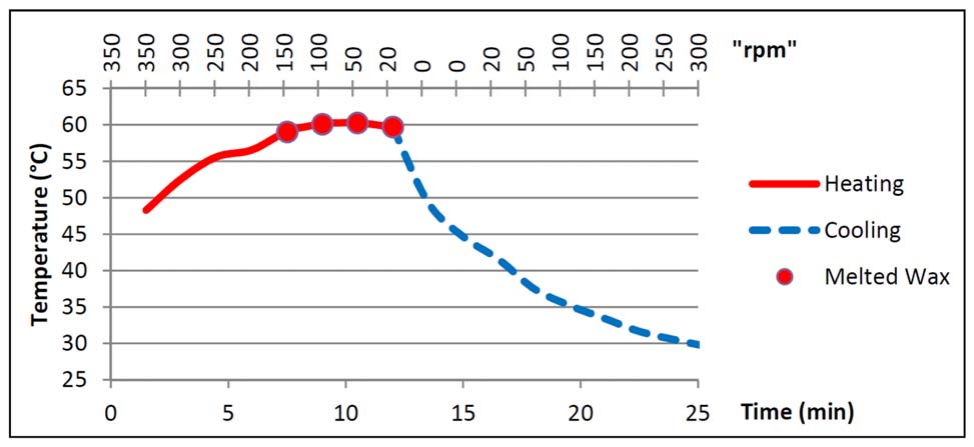
Microfluidic CD heating profile. Heating profile for Microfluidic CD and wax melting points.

In figure 6 we show the heating profile for the microfluidic CD during the heating process. The x-axis represents the temperature of the microfluidic CD surface, while the y-axis presents the experiment time (bottom y-axis) and the CD spinning speed (top y-axis). The forced convection heating is fixed at 130°C for this experiment.

The graph can be divided up into two main parts: the first part represents the heating profile of the CD (heater is ON), while the second part represents the cooling profile of the CD (heater is OFF). The CD surface temperature increases dramatically in the first 2 minutes from room temperature at 27°C to approximately 48°C, then continues to rise somewhat slower to the minimum wax melting temperature of 57.2°C (135°F), and peaks out around 60°C at around the 8th minute mark. The temperature is observed to remain around 60°C for the next 4 minutes. The forced convection heating is then shut off, and the CD spinning is also stopped temporarily before the start of the cooling process. Next, the CD is left to cool at increasing rpm speeds and the temperature reaches room temperature in 12 minutes.

Throughout the experiment, we had observed that the temperature of the CD surface outside of the heating zone rises slightly, and saturates at around 5°C above room temperature. It is also determined experimentally that the heat setting of 130°C provides the optimum balance between the shortest time to melt the wax plugs without exposing the PMMA material to heat shock (where sudden temperature increase melts or deforms the PMMA material).

The result provides an understanding of how the CD surface responds to forced convection heating. It is clear that with the forced convection heating set at 130°C, the CD would require 8 minutes to melt the wax to operate the VCV.

### 2. Vacuum/Compression Valve (VCV) Effectiveness

**Figure 7:**
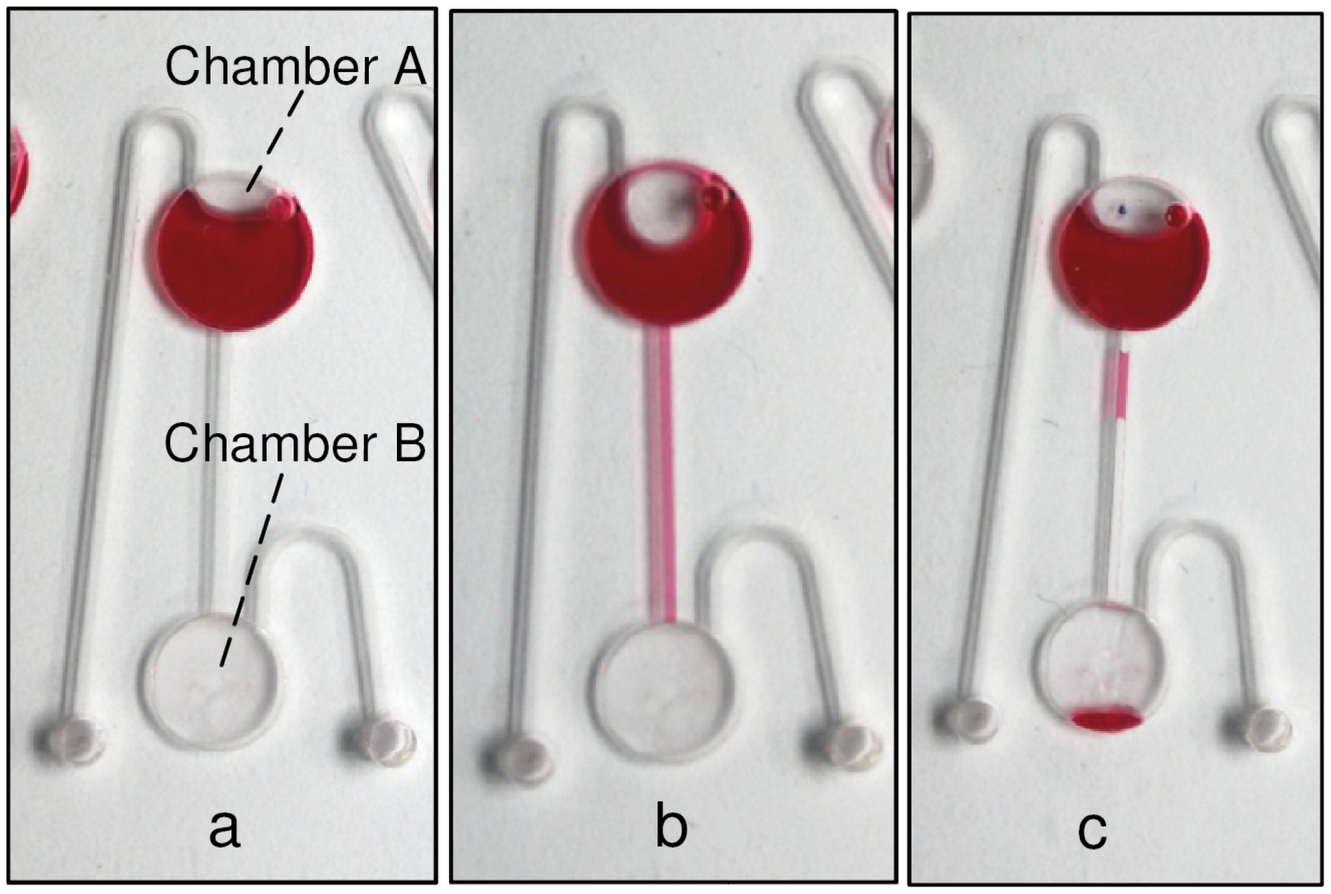
Liquid postion during testing process. Liquid state during the testing of VCV valving effectiveness (a) initial liquid postion during low spinning speed, (b) liquid goes into the channel at spinning frequency >900 rpm, (c) final position of liquid at 1500 rpm.

**Figure 8:**
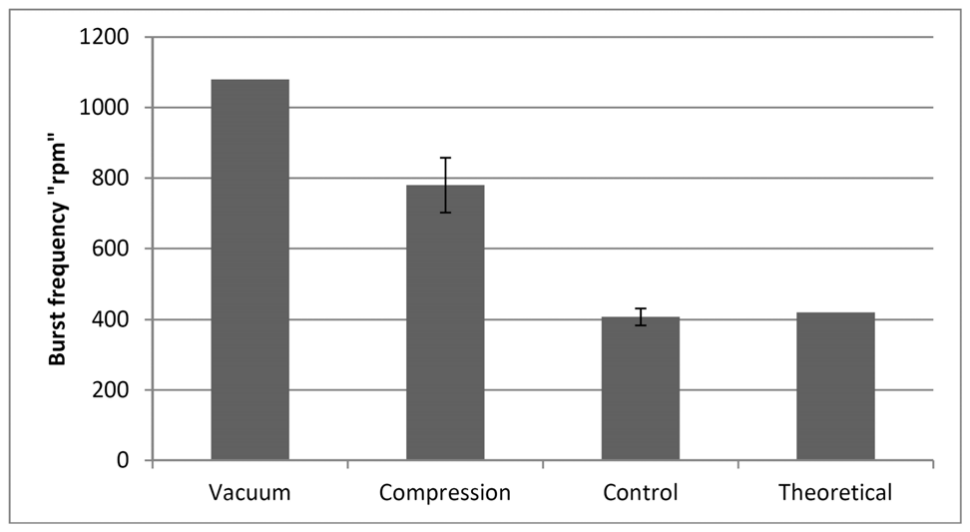
Burst frequency for VCV valve. Burst frequency "rpm" for liquid using the proposed Vacuum/Compression valaving, and burst frequency for Control & Theoretical calculation.

The second part of this study focuses on testing the effectiveness of the proposed valve and its response towards increasing pressure produced by an incremental spinning speed. Figure 7 presents the liquid behavior at three different points of the experimental test for the air-compression state (venting hole B is sealed). Figure 7(a) presents the initial status of the liquid before and during the early stages of spinning, figure 7(b) shows liquid spilling into the micro-channel due to the high spinning speed (more than 900 rpm) that leads to air compression inside the destination chamber. Figure 7(c) shows the final result at 1500 rpm where a minor leakage of one droplet occurs when the liquid/compressed-air interface destabilizes and some air escapes in the form of bubbles up the micro-channel. This result shows that the proposed VCV is able to prevent liquid from bursting into the destination chamber up to speeds of 1000 rpm. However, at spinning speeds above 1000 rpm, although the fluid still does not flow into Chamber B, some leakage is observed. Experimental data for compression and vacuum valving is presented separately in figure 8. Moreover, the experimental results are compared to the control (experimental results without valving) and theoretical results calculated using Equations (1) and (2).

From our results, it is clear that vacuum valving (source chamber venting hole sealed) is more dynamic than compression valving (destination chamber venting hole sealed). There are two reasons for this; the first is the smaller volume of air in vacuum valving (air that is trapped in the source chamber on top of the liquid and inside the venting channel), is hard to expand. The second is the effect of the lower centrifugal force experienced by the liquid in the source chamber with a vacuum valve. In comparison to the liquid in the micro-channel in the configuration with a compression valve, the liquid in a configuration with a vacuum valve is closer to the center of the CD.

In comparison to the wax valving proposed by Allen et al [18] and Abi-Samra et al [19], the proposed VCV method prevents any mixing between the sealing material and the test samples. This design improvement gives way to the possibility of using different types of material for valving which may be more easily managed. Moreover, the layout of the VCV on the CD can be easily relocated to be further away from the test samples. This is an advantage when compared to the valving method by Abi-Samra et al [19] where the heating source is directly focused on the micro-channels, concurrently heating the wax and the liquid in the channel.

### 3. Applications of VCV

**Figure 9:**
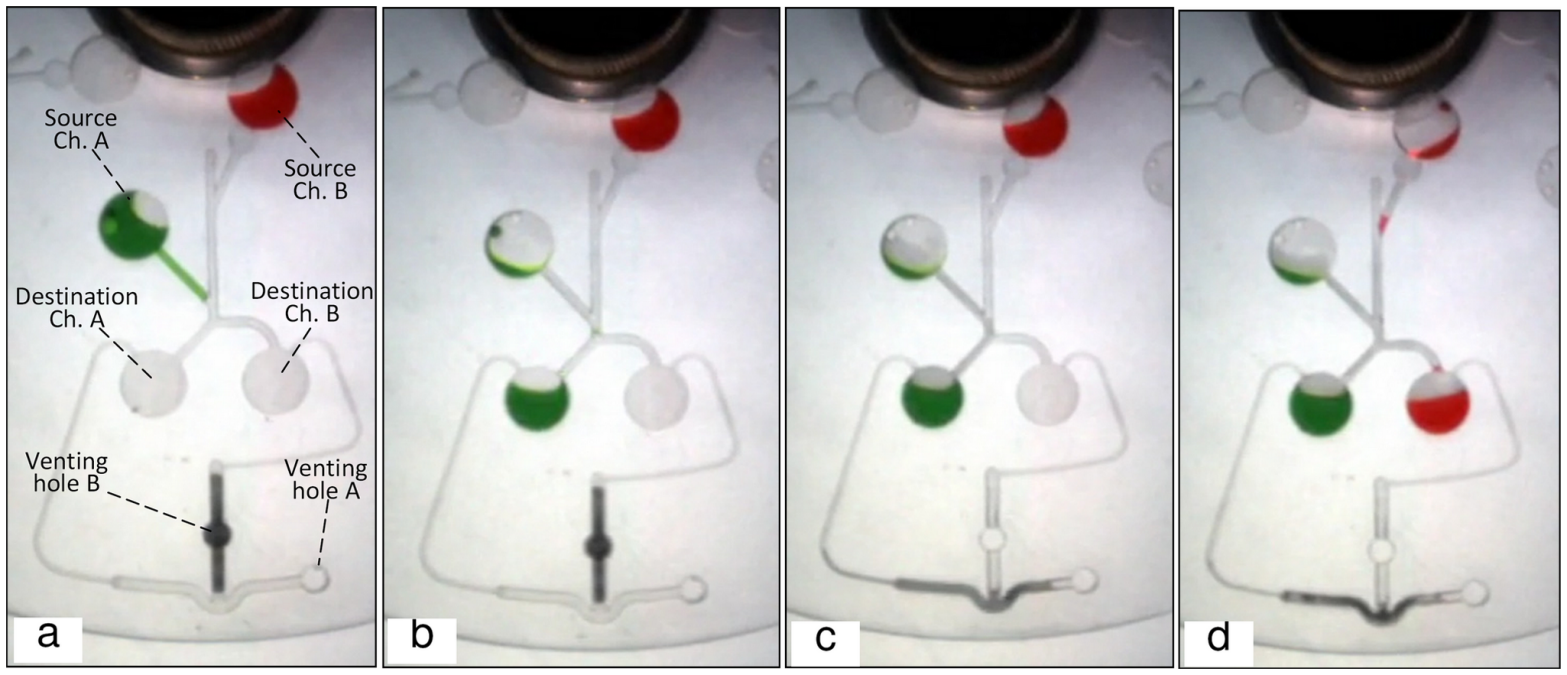
Liquid switching sequence. Experment sequence for liquid switching using VCV valving method, (a) green clored liquid flows out from source chamber A, (b) green liquid switched to chamber A due to the compressed air in chamber B, (c) venting hole B released by melting wax plug, and wax moves to block venting hole A, (d) red liquid switched to chamber B due to the air compression in chamber A.

**Figure 10:**
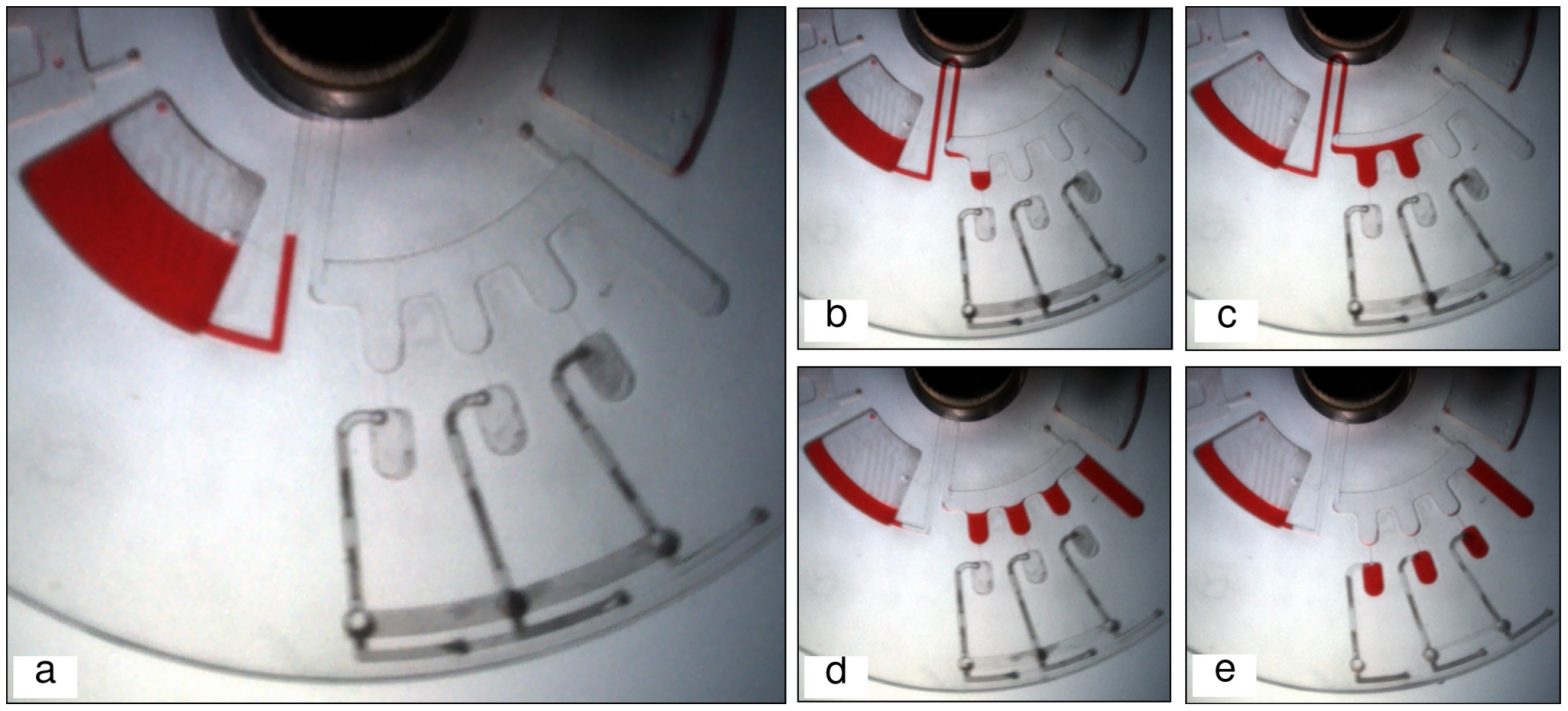
Liquid metering sequence. Experment sequence for liquid metering using VCV valving method, (a) liquid pumped from source chamber to the metering chambers, (b) liquid filling the first metering chamber, (c) liquid filled the second metering chamber and moving to the third, (d) liquid filled all metering chambers and overflowed to the waste chamber, (e) wax-plug melted and liquid moved to the destination chambers.

In figures 9 and 10 we show photos from two types of applications at various stages during the tests. Figure 9(a) shows the liquid bursting out of source chamber A at 360 rpm. It is observed that at the junction of the two channels, although the perpendicular angle of the channel leading from the junction towards destination chamber B helps to direct liquid flow into destination chamber A, initially a very small volume of liquid enters the start of the channel leading into chamber B. This causes the trapped air in chamber B to be compressed, and the compressed air then pushes the liquid back out of the channel into the junction. The small volume of liquid then flows with the rest of the liquid towards destination chamber A (figure 9(b)). When the liquid from source chamber A completely enters destination chamber A, the forced convection heating is turned ON to melt the wax plug and to release venting hole A (figure 9(c)). The centrifugal force pushes the molten wax towards venting hole B and seals it. The heating source is then turned OFF and the spinning speed is increased gradually. At 650 rpm, the liquid from source chamber B bursts. Similarly to the case in figure 9(b), any liquid that enters into the channel leading towards destination chamber A creates air-compression, and the liquid is pushed back out into the junction. The liquid from chamber B is observed to flow toward destination chamber B (figure 9(d)).

The result shows that switching with a VCV can be controlled accurately, cleanly, and at low spinning speed. This is advantageous when compared to switching processes applying external air pressure by Kong et al [17], and Coriolis force by Kim et al [13]. Moreover, by applying more VCVs, liquid flow switching into more than two destination chambers can be accomplished.

Figure 10 presents the experimental sequence for the microfluidic metering process. As shown, the liquid is pumped from the source chamber to the metering chambers (figure 10(a)). Then, liquid starts to flow and fill the three metering chambers without entering the destination chamber because of the compressed air (figure 10 (b & c)). After all metering chambers are filled; the extra liquid flows into the waste chamber (figure 10(d)). The heating source is turned ON to heat the CD surface to 60°C (which is the melting temperature for the wax plugs). Once the wax-plug has been melted away, the venting hole is opened and the liquid bursts from the metering chambers into the destination chambers (figure 10(e)).

One additional observation made during the melting of the wax, particularly during the initial preparation of the wax plug, is the spreading of the wax into unintended areas. While the VCV is designed such that melted wax flows away from the liquid chambers during spinning, care needs to be taken when loading melted wax over the venting holes. Because wax is hydrophilic on PMMA surfaces, melted wax easily seeps into any channel it comes in contact with. To prevent this, only a precise volume of wax (sufficient to cover the venting hole) needs to be injected into the venting hole such that there will be no excess wax flowing into the connected channel. However, in our experiments, the melted wax does not enter the liquid chamber area and thus does not affect the outcome of the experiments.

The presented microfluidic metering process has many advantages in comparison to published metering method introduced by Mark et al [26], [27]. Mark et al’s [27] method requires high spinning frequency to generate the turbulence at the air-liquid contact point for the liquid to burst into the destination chambers. In contrast, our proposed metering process can be performed at low spinning speeds (less than 400 rpm). Furthermore, the destination chamber for our proposed method can be connected to other microfluidic networks on the CD (where other processes can be performed) by implementing the VCV at appropriate points.

### Conclusion

This paper proposes VCV valving on microfluidic CDs using paraffin wax to seal venting chambers/holes. The results indicate high flexibility and accuracy in controlling the liquid burst frequency. It is noticed that a vacuum valve on the source chamber is more resilient against bursting at high spinning frequencies compared to compression valves. Furthermore, the presented VCV method can reduce the direct heating of samples and reagents in the microfluidic process.

Two microfluidic processes which are liquid flow switching and liquid metering have been implemented with the VCV, and have been successfully demonstrated. The experimental results show that by using the VCV valving, the required spinning frequency to perform the process is reduced greatly, and the VCV valving allows for multiple path switching.

In the liquid flow switching demonstration, we have also showed a novel way of implementing two valves using a single VCV wax plug. This is done by positioning the valves such that melted wax from a normally-closed valve (which releases it when the wax is melted) is later transferred to a normally-opened valve (which seals it when the wax solidifies) by centrifugal force.
